# Supplementary material for: Nanoparticle-Mediated Delivery of Deferasirox: A Promising Strategy Against Invasive Aspergillosis
Source: Bioengineering (Basel). 2024 Nov 5;11(11):1115. doi: 10.3390/bioengineering11111115 (PMC11591955; doi:10.3390/bioengineering11111115)
Supplement: Supplementary file 1 [file bioengineering-11-01115-s001.zip › bioengineering-3263820-supplementary.pdf]

bioengineering

MDPI

Supplementary Data

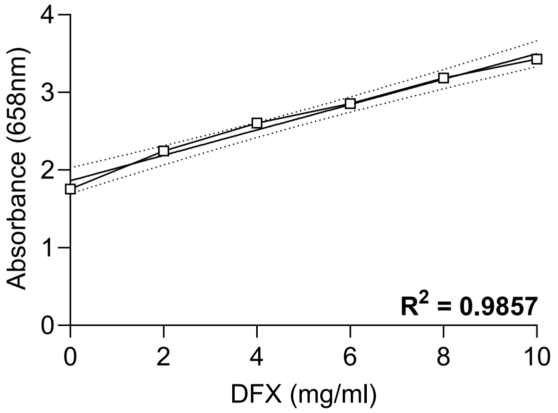

Figure S1. Deferasirox (DFX) standard curve in fetal bovine serum (absorbance measured at 658 nm).

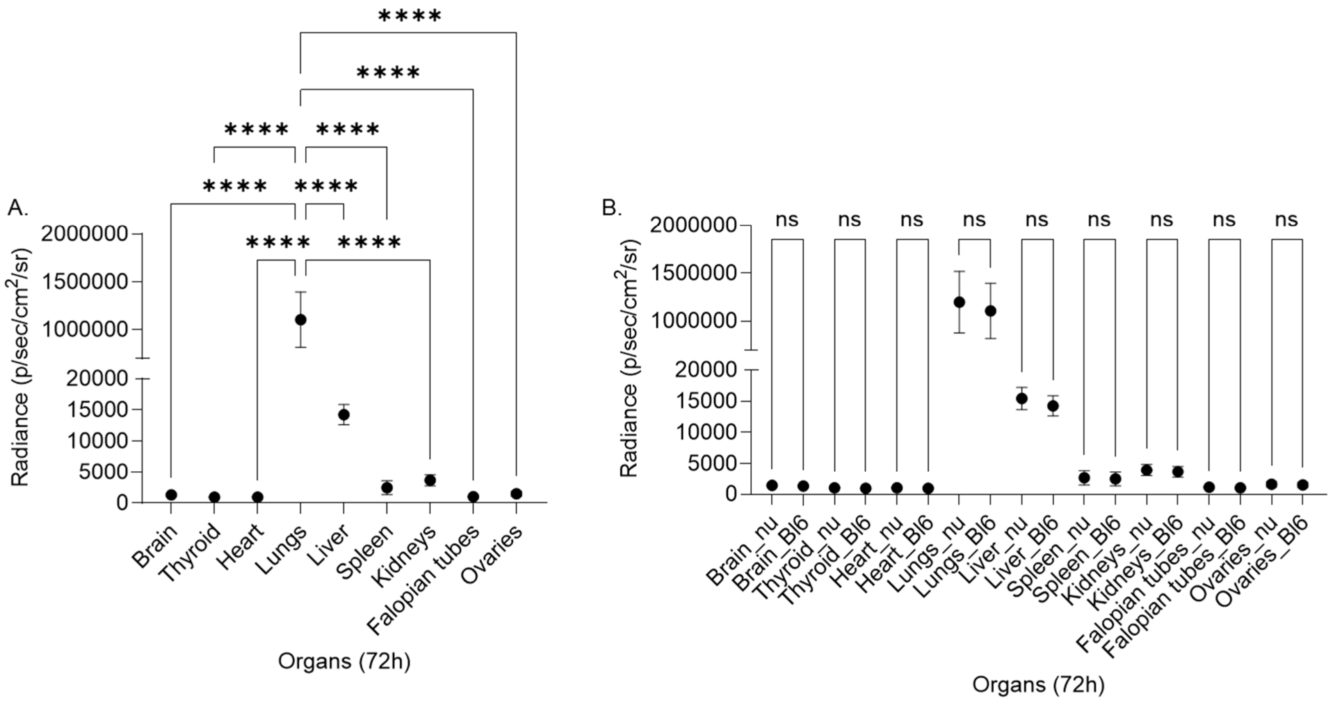

Figure S2. Biodistribution study in C57Bl/6 mice. A. Quantification of indocyanine green (ICG) signal in B57Bl/6 organs after 72h (**** p <0.00001). B. Comparison of ICG signal detected in C57Bl/6 and nu/nu mice (ns = not significant).

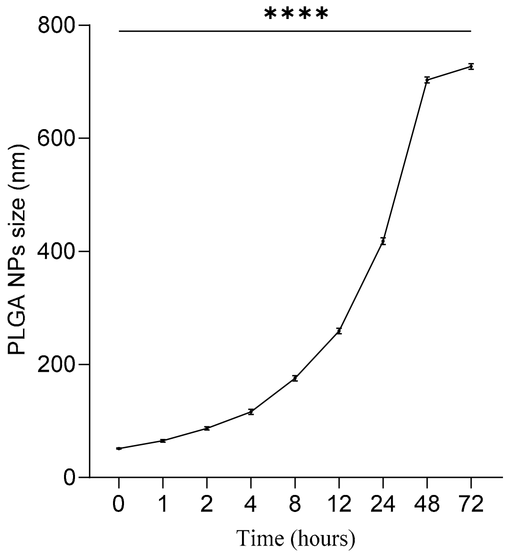

Figure S3. DFX PLGA NP size monitoring in FBS pH 7.4 via DLS measurements. NP size was significantly increased in FBS after 72 h. t = 0 h size 51.46 nm; t = 72h size 726.8 nm. (**** $p < 0.00001$).

Disclaimer/Publisher's Note: The statements, opinions and data contained in all publications are solely those of the individual author(s) and contributor(s) and not of MDPI and/or the editor(s). MDPI and/or the editor(s) disclaim responsibility for any injury to people or property resulting from any ideas, methods, instructions or products referred to in the content.
